# Zonisamide Enhances Motor Effects of Levodopa, Not of Apomorphine, in a Rat Model of Parkinson's Disease

**DOI:** 10.1155/2018/8626783

**Published:** 2018-12-18

**Authors:** Haruo Nishijima, Yasuo Miki, Shinya Ueno, Masahiko Tomiyama

**Affiliations:** ^1^Department of Neurology, Aomori Prefectural Central Hospital, Aomori, Japan; ^2^Department of Neurophysiology, Institute of Brain Science, Hirosaki University Graduate School of Medicine, Hirosaki, Japan; ^3^Department of Neuropathology, Institute of Brain Science, Hirosaki University Graduate School of Medicine, Hirosaki, Japan

## Abstract

Zonisamide is a relatively recent drug for Parkinson's disease. Multiple hypotheses have been proposed to explain the antiparkinsonian effects of zonisamide. However, it is still unclear whether the effect of zonisamide is mainly due to dopaminergic modification in the striatum, or if zonisamide works through nondopaminergic pathways. We conducted the present study to determine the mechanism that is mainly responsible for zonisamide's effects in Parkinson's disease. We examined the effects of zonisamide on motor symptoms in a hemiparkinsonian rat model when administered singly, coadministered with levodopa, a dopamine precursor, or apomorphine, a D1 and D2 dopamine receptor agonist. We used 6-hydroxydopamine-lesioned hemiparkinsonian rats, which were allocated to one of five groups: 14 rats received levodopa only (6 mg/kg), 12 rats received levodopa (6 mg/kg) plus zonisamide (50 mg/kg), six rats received apomorphine only (0.05 mg/kg), six rats received apomorphine (0.05 mg/kg) plus zonisamide (50 mg/kg), and six rats received zonisamide only (50 mg/kg). The drugs were administered once daily for 15 days. We evaluated abnormal involuntary movement every 20 min during a 3 h period following the injection of drugs on treatment Days 1, 8, and 15. Western blot analyses for dopamine decarboxylase and vesicular monoamine transferase-2 were performed using striatal tissues in the lesioned side of rats in the levodopa only group (*n* = 6) and levodopa plus zonisamide group (*n* = 4). Levodopa-induced abnormal involuntary movement was significantly enhanced by coadministration of zonisamide. In contrast, zonisamide had no effect on apomorphine-induced abnormal involuntary movement. Zonisamide monotherapy did not induce abnormal involuntary movement. Zonisamide did not affect striatal expression of dopamine decarboxylase or vesicular monoamine transferase-2. In conclusion, zonisamide appears to generate its antiparkinsonian effects by modulating levodopa-dopamine metabolism in the parkinsonian striatum.

## 1. Introduction

Parkinson's disease (PD) is a neurodegenerative disease characterized by motor symptoms such as tremor, akinesia, hypokinesia, rigidity, and postural disturbance [1]. The most effective treatment for PD is dopamine replacement therapy using the dopamine precursor, L-3,4-dihydroxyphenylalanine (levodopa, L-dopa) [2]. Other drugs are also used to treat PD, including dopamine agonists, catechol-O-methyltransferase inhibitor, monoamine oxidase-B inhibitor, *N*-methyl-D-aspartate receptor antagonist (amantadine), and adenosine A2A receptor antagonist (istradefylline) [3]. 1,2-Benzisoxazole-3-methaesulfonamide (zonisamide) is an antiparkinsonian drug developed relatively recently. It has been primarily used as an antiepileptic drug and has been reported to be efficacious in the treatment of PD [4]. The beneficial effects of zonisamide in PD were serendipitously found in a patient who had epilepsy and PD [4]. Subsequent clinical research has revealed that a relatively low dose of zonisamide (25–50 mg/day), compared with the dose for epilepsy (200–600 mg/day), is efficacious for improvement of motor symptoms in advanced PD patients receiving levodopa treatment [5–7]. Now, zonisamide is approved as an adjunctive treatment in patients with advanced PD who show insufficient response to levodopa treatment. Moreover, recently, it has been reported that zonisamide monotherapy is effective for treatment in de novo patients with early PD [8].

The precise mechanisms of zonisamide for PD are not clearly understood yet. Multiple hypotheses have been proposed to explain the antiparkinsonian effects of zonisamide [9, 10]; however, it is still unclear whether the effect of zonisamide is mainly due to dopaminergic modification in the striatum, or if zonisamide works through nondopaminergic pathways.

A 6-hydroxydopamine- (6-OHDA-) lesioned hemidopamine denervated rat is a well-established animal model of PD. This rat responds very sharply to dopaminergic stimulation in the lesioned striatum and presents marked abnormal involuntary movements (AIMs) in its body contralateral to the lesion [11, 12]. Behavioral analysis of this animal model may shed light on the mechanisms of zonisamide. We conducted the present study to determine the mechanism that is mainly responsible for zonisamide's effects in PD. The aim of the present study is to examine the effects of zonisamide on motor symptoms in a hemiparkinsonian rat when administered singly, coadministered with levodopa, a dopamine precursor, or apomorphine, a D1 and D2 dopamine receptor agonist.

## 2. Materials and Methods

### 2.1. Animals

We used 44 male Wistar rats (CLEA Japan Inc., Tokyo, Japan) in this study. The experimental procedures complied with the “Principles of Laboratory Animal Care” (NIH Publication Vol 25, No. 28 revised 1996; http://grants.nih.gov/grants/guide/notice-files/not96-208.html) and the guidelines for animal research issued by the Physiological Society of Japan and by Hirosaki University School of Medicine. All efforts were made to minimize the number of animals used and their suffering.

### 2.2. Surgery to Create Hemiparkinsonian Rats

At 10 weeks of age, all the rats underwent stereotactic infusion of 6-OHDA (Sigma, San Diego, CA, USA) into the medial forebrain bundle on the right side. The rats were pretreated with desipramine (25 mg/kg, intraperitoneally) (Sigma) 30 min before the injection of 6-OHDA to prevent the denervation of noradrenergic neurons. A stainless steel needle (0.4 mm diameter) was inserted through a small burr hole on the right side of the skull, and the needle tip was placed in the right medial forebrain bundle (4.5 mm posterior to the bregma, 1.2 mm lateral to the sagittal suture, and 8.5 mm ventral to the periosteum surface) according to the atlas of Paxinos and Watson [13]. We injected 6-OHDA (8 *µ*g/4 *µ*L in saline with 0.01% ascorbic acid) over 4 min. After injection, the needle was left in place for 2 min to prevent backflow leakage from the injection site. To evaluate the extent of dopaminergic denervation, 2 weeks after the 6-OHDA injection, the rats were challenged with an apomorphine (Sigma) injection (in saline with 0.1% ascorbic acid, 0.05 mg/kg, subcutaneously). Rats that made more than 20 contralateral (to the left) turns during a 5 min period between 15 and 20 min after the apomorphine injection, indicating a lack of dopaminergic function in the striatum, were considered to be a model of PD and were included in the present study. We have previously shown that rats meeting this criterion have lost more than 99% of the dopamine in their striatum [14].

### 2.3. Drug Treatment

Seven weeks after the surgery (5 weeks after the apomorphine test), the rats were randomly allocated to one of five groups: 14 rats received levodopa methyl ester only (6 mg/kg, intraperitoneal injection) (Sigma), 12 rats received levodopa (6 mg/kg) plus zonisamide (50 mg/kg, intraperitoneal injection), six rats received apomorphine only (0.05 mg/kg, subcutaneous injection), six rats received apomorphine (0.05 mg/kg) plus zonisamide (50 mg/kg), and six rats received zonisamide only (50 mg/kg). Rats in the levodopa only group and the levodopa plus zonisamide group also received benseraside (10 mg/kg, intraperitoneal injection) (Sigma) at the same time as the levodopa injection to prevent peripheral decarboxylation of levodopa. Zonisamide was provided by Sumitomo Dainippon Pharma Co., Ltd., Osaka, Japan. All drugs were administered once daily for 15 days.

### 2.4. Behavioral Analyses

An AIM score [11] was measured every 20 min during the 3 h period following the injection of levodopa (nine times) on treatment Days 1, 8, and 15. Scores were based on the duration and persistence of involuntary purposeless behavior during the 1 min observation period. AIMs were classified into four subtypes: locomotive dyskinesia, axial dystonia, limb dyskinesia, and orolingual dyskinesia (see video links published in [15]). For each of the four subtypes, each rat was scored on a scale from 0–4: 1 = occasional; 2 = frequent; 3 = continuous but interrupted by sensory distraction; and 4 = continuous, severe, and not interrupted by sensory distraction [11]. The observer who scored the AIM (MT) was blind to the treatment condition. It has been shown that, among the four subtypes of AIMs in 6-OHDA-lesioned levodopa-treated rats, three subtypes, axial dystonia, limb dyskinesia, and orolingual dyskinesia, are equivalent to levodopa-induced dyskinesia (LID) in patients with PD [12, 16, 17]. In contrast, locomotive dyskinesia has been reported to be induced by not only levodopa but also by long-acting dopamine agonists [17, 18], and thus does not provide any specific measure of levodopa-induced motor complications [12, 16, 17]. Thus, the total score of axial dystonia, limb dyskinesia, and orolingual dyskinesia (ALO AIM score) has been used as an index of severity of LID.

### 2.5. Western Blot Analysis

Rats were sacrificed by decapitation after AIM scoring on treatment Day 15. Brains were dissected out and frozen rapidly at −70°C. For the present study, the dopamine-denervated striatum of rats treated with levodopa only (*n* = 6) and rats treated with levodopa plus zonisamide (*n* = 4) were subjected to immunoblotting. Western blot analysis was performed as described previously [19]. Rabbit polyclonal anti-dopa decarboxylase (DDC) (PAB9598; Abnova, Taipei, Taiwan; 1 : 1,000), anti-vesicular monoamine transporter-2 (VMAT-2) (20873-1-AP; Proteintech, Chicago, IL; 1 : 1,000), and anti-actin (A2066; Sigma, St. Louis, MO; 1 : 1,000) antibodies were used as the primary antibodies. A semiquantitative analysis of protein levels was performed using the Image J software provided by the National Institutes of Health.

### 2.6. Data Analysis

Statistical analyses were performed using the computer software program Ekuseru-Toukei 2015 (Social Survey Research Information Company, Ltd., Tokyo, Japan) and Excel (Microsoft Corporation, Redmond, WA, USA). All data were expressed as mean ± standard deviation. The 3 h sum score of the AIM of each subtype and the total score of axial dystonia, limb dyskinesia, and orolingual dyskinesia (ALO AIM score) were analyzed. AIM scores at each time point on treatment Day 15 were also analyzed. Differences of AIM scores were examined by two-way repeated measures ANOVA with the post hoc Scheffe test. Values of the western blot analyses were examined using a two-sample *t*-test. A probability level of less than 5% (*p* < 0.05) was considered statistically significant.

## 3. Results

### 3.1. 3 h Total Score of AIM on Days 1, 8, and 15

**Figure 1:**
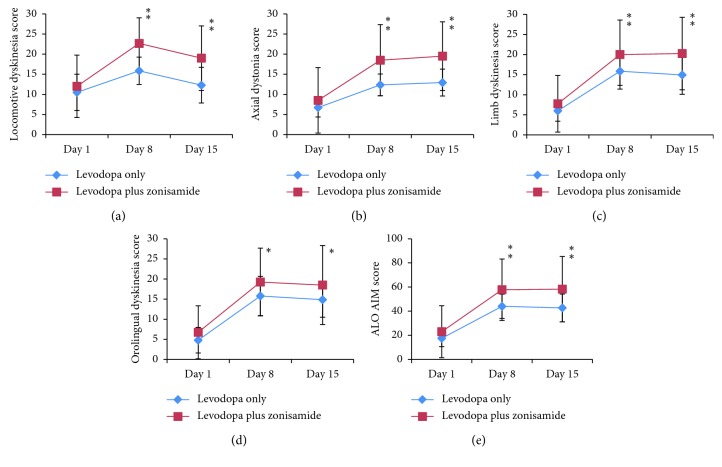
The 3 h totals of abnormal involuntary movement (AIM) scores on treatment Days 1, 8, and 15 in the levodopa only group and levodopa plus zonisamide group. AIM scores are shown for four subtypes: locomotive dyskinesia (a), axial dystonia (b), limb dyskinesia (c), and orolingual dyskinesia (d) and for the total score of axial dystonia, limb dyskinesia, and orolingual dyskinesia (ALO AIM) (e). Diamonds indicate the scores of the levodopa only group, and squares indicate the scores of the levodopa plus zonisamide group. Data are expressed as mean ± standard deviation. *∗p* < 0.05; *∗∗p* < 0.01 compared with the levodopa only group.

**Figure 2:**
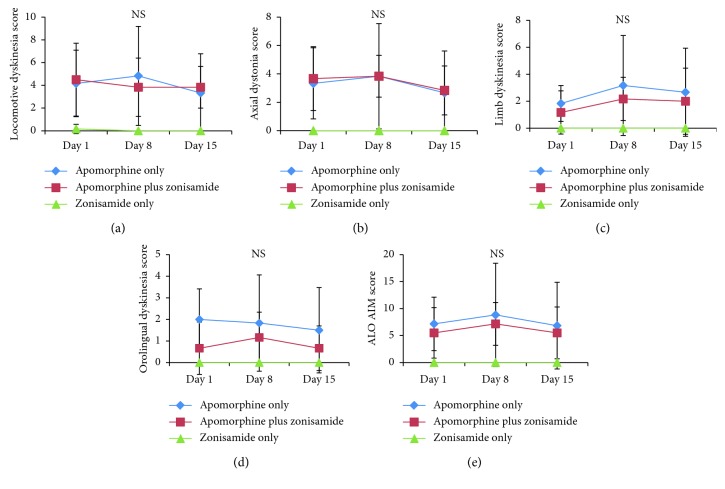
The 3 h totals of abnormal involuntary movement (AIM) scores on treatment Days 1, 8, and 15 in the apomorphine only group, apomorphine plus zonisamide group, and zonisamide only group. AIM scores are shown for four subtypes: locomotive dyskinesia (a), axial dystonia (b), limb dyskinesia (c), and orolingual dyskinesia (d), and for the total score of axial dystonia, limb dyskinesia, and orolingual dyskinesia (ALO AIM) (e). There were no significant differences between the apomorphine only group and apomorphine plus zonisamide group. Diamonds indicate the scores of the apomorphine only group, squares indicate the scores of the apomorphine plus zonisamide group, and triangles indicate the scores of the zonisamide only group. Data are expressed as mean ± standard deviation. NS, not significant.

On treatment Day 1, zonisamide showed no effect on AIMs induced by either levodopa (Figure 1) or apomorphine (Figure 2). On Day 8 and Day 15, AIM scores of all categories were significantly higher in the levodopa plus zonisamide group when compared with the levodopa only group (Figure 1). Zonisamide treatment had no impact on apomorphine-induced AIMs throughout the experiment (Figure 2). Zonisamide monotherapy induced no AIMs (Figure 2).

### 3.2. Scores at Each Time Point after Drug Injection on Day 15

**Figure 3:**
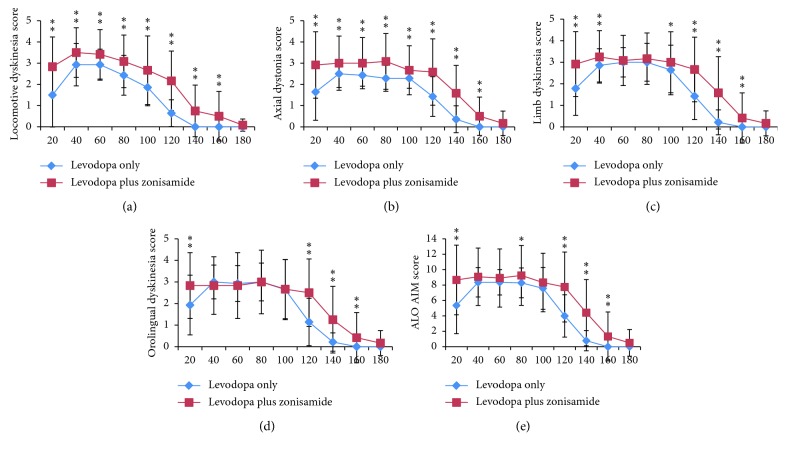
Abnormal involuntary movement (AIM) scores at each time point after drug injection on treatment Day 15 in the levodopa only group and the levodopa plus zonisamide group. AIM scores are shown for four subtypes: locomotive dyskinesia (a), axial dystonia (b), limb dyskinesia (c), and orolingual dyskinesia (d), and for the total score of axial dystonia, limb dyskinesia, and orolingual dyskinesia (ALO AIM) (e). Diamonds indicate the scores of the levodopa only group and squares indicate the scores of the levodopa plus zonisamide group. Data are expressed as mean ± standard deviation. *∗p* < 0.05; *∗∗p* < 0.01 compared with the levodopa only group.

**Figure 4:**
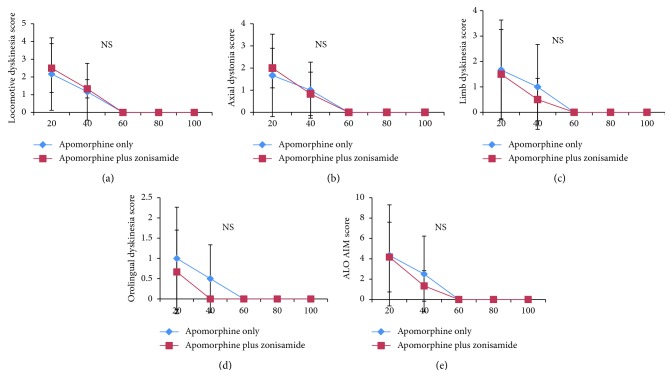
Abnormal involuntary movement (AIM) scores at each time point after drug injection on treatment Day 15 in the apomorphine only group and the apomorphine plus zonisamide group. AIM scores are shown for four subtypes: locomotive dyskinesia (a), axial dystonia (b), limb dyskinesia (c), and orolingual dyskinesia (d), and for the total score of axial dystonia, limb dyskinesia, and orolingual dyskinesia (ALO AIM) (e). There were no significant differences between the groups. Diamonds indicate the scores of the apomorphine only group and squares indicate the scores of the apomorphine plus zonisamide group. Data are expressed as mean ± standard deviation. NS, not significant.

On Day 15, in the levodopa plus zonisamide group, locomotive dyskinesia scores at 20 min to 160 min after drug injection were significantly higher when compared with those of the levodopa only group (Figure 3(a)). ALO AIM scores in the levodopa plus zonisamide group were also significantly higher at 20, 80, 120, 140, and 160 min after drug injection when compared with those of the levodopa only group (Figure 3(e)). Scores reflecting severity of peak-dose dyskinesia (ALO AIM scores at 60–100 min [12]) were slightly higher at 80 min only (Figure 3(e)). There were no significant differences in any category at any time point on Day 15 between the apomorphine plus zonisamide group and the apomorphine only group (Figure 4).

### 3.3. Western Blot Analysis

**Figure 5:**
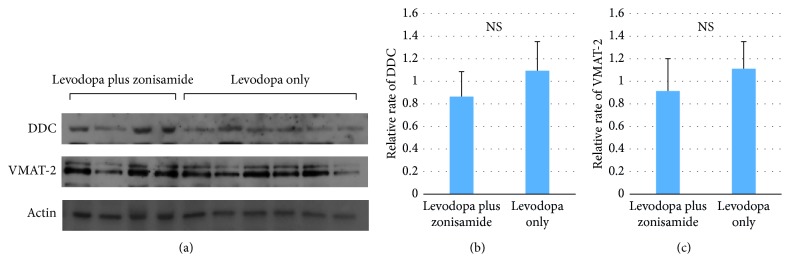
Western blot analysis of the dopamine-denervated striatum in rats treated with levodopa plus zonisamide (*n* = 4) and levodopa only (*n* = 6). The raw data for dopamine decarboxylase (DDC) and vesicular monoamine transferase-2 (VMAT-2) (a). There were no significant differences in the levels of DDC (b) and VMAT-2 (c) relative to the levels of actin between the two groups. NS, not significant.

We performed western blot analysis to investigate the expression levels of DDC and VMAT-2 in the lesioned striatum. There was no significant difference of these proteins between the levodopa only group and the levodopa plus zonisamide group (Figure 5).

## 4. Discussion

In the present study, zonisamide significantly increased levodopa-induced AIMs. In the time course analyses on treatment Day 15, it appeared that zonisamide enhanced and prolonged the motor effects of levodopa, although peak-dose dyskinesia got slightly worsened. In contrast, zonisamide had no impact on apomorphine-induced AIMs. Zonisamide monotherapy induced no AIMs. Thus, zonisamide appears to elicit beneficial effects for PD through modification of levodopa-dopamine metabolism in the striatum.

Our result is in agreement with a previous report that zonisamide enhances levodopa-induced rotational behavior in 6-OHDA-lesioned parkinsonian rats [20]. However, Oki et al. have recently reported that zonisamide decreases levodopa-induced dyskinesia-like behavior in the same rat model of PD, although the effect was minimal [21]. In their experiment, the levodopa dose was 12 mg/kg twice a day, which is four times the dose per day that was used in our experiment. Inconsistency between the studies may be due to the drug dose, and further examinations using various drug doses are required to resolve this issue.

When used at the therapeutic dose for epilepsy, zonisamide has been reported to increase dopamine concentration in normal rat striatum [22, 23]. Previously proposed mechanisms of the zonisamide-induced increase in dopamine concentration are activation of tyrosine hydroxylase [9] and inhibition of monoamine oxidase (MAO)-B [24, 25]. However, tyrosine hydroxylase is not involved in levodopa metabolism, and MAO-B has a minor role in dopamine oxidation in the rat brain [26, 27]. Thus, neither mechanism can fully explain the enhanced levodopa effect in the present study. In clinical situations, MAO-B inhibition apparently has beneficial effects on PD symptoms [28]. Clinical effects of zonisamide may be partly due to MAO-B inhibition; however, the results of the present study using a rodent model may not be explained by reduced activity of MAO-B. A recent study using cultured cells from mice has shown that zonisamide can inhibit both MAO-A and MAO-B [29]. MAO-A inhibition may explain the present result, although further studies using parkinsonian model would be required to support this idea.

Zonisamide increases the extracellular dopamine concentration after levodopa administration in the dopamine-denervated striatum in a rat model of Parkinson's disease [30]. It has also been shown that 3,4-dihydroxyphenylacetic acid, a metabolite of dopamine, increases with co-administration of zonisamide and levodopa when compared with levodopa alone, although this effect was not seen in all of the parkinsonian rats [20]. Our western blot analyses showed no change in striatal expression of DDC or VMAT-2, suggesting that dopamine release may not be responsible for the dopaminergic effect of zonisamide. Inhibition of reuptake of levodopa-derived dopamine is another possible mechanism of the dopaminergic effect of zonisamide. In the dopamine-denervated striatum, extracellular dopamine is uptaken by the norepinephrine transporter, serotonin transporter, organic cation transporter-3, and plasma membrane monoamine transporter [31]. These transporters could be targets of zonisamide in its beneficial effects for PD. Whether functions of these transporters are modified by zonisamide should be examined in the future studies.

There are some limitations of the present study. First, the behavioral assessment focused only on dyskinesia-like movements. Using the AIM scoring method, it is difficult to distinguish improvement of akinesia from induction of dyskinesia in a rodent model of PD. Evaluation of the effect on motor symptoms of PD using other behavioral testing, such as the cylinder test, forepaw steps test, and rotarod test, is warranted to gain a more comprehensive understanding of the drug effects on behavior. Moreover, future studies using a primate model are required to confirm the effect of zonisamide. Second, striatal dopamine levels after drug treatment were not measured in the present study. To support our idea that zonisamide alters the striatal dopamine levels after levodopa administration, further studies are required with direct measurement of dopamine levels in the striatum with our animal PD model and experimental settings.

In conclusion, zonisamide appears to elicit its antiparkinsonian effects by modulating levodopa-dopamine metabolism in the striatum.

## Data Availability

All the raw data used to support the findings of this study are available from the corresponding author upon request.
